# Fe-doped polydopamine pickering emulsion as a nanoplatform for synergistic photothermal-ferroptosis immunotherapy in breast cancer

**DOI:** 10.3389/fphar.2026.1956784

**Published:** 2026-10-07

**Authors:** Ling-Yu Xu, Peng Zhang, Min-Jing Yu, Hao-Ran Li, Li-Chen Cao, Haiyan Gao

**Affiliations:** 1 Department of Breast Surgery, Changzhou Cancer Hospital (The Fourth People’s Hospital of Changzhou), Changzhou, China; 2 School of Materials Science and Engineering, Changzhou University, Changzhou, China

**Keywords:** fe-doped polydopamine, ferroptosis, photothermal therapy, pickering emulsion, tumor immunotherapy

## Abstract

**Introduction:**

Triple-negative breast cancer remains a formidable clinical challenge owing to its immunosuppressive microenvironment and inherently low immunogenicity.

**Methods:**

To address this, we developed a multifunctional Pickering emulsion (Fe-PDA-PE-R837) stabilized by iron-doped polydopamine (Fe-PDA) nanoparticles, which function dually as photothermal agents and localized iron donors, while the soybean oil core encapsulates the immunoadjuvant R837. The Fe-PDA nanoparticles exhibited uniform spherical morphology, excellent colloidal stability, and a broad absorption band extending to the NIR region. Upon 808 nm laser irradiation, the Fe-PDA shell generates local hyperthermia with a photothermal conversion efficiency of 38.5%, disrupts intracellular iron homeostasis to provoke ferroptotic cell death, as evidenced by GSH depletion and MDA accumulation, while the stimuli-responsive release of R837 coupled with immunogenic cell death (ICD) elicits potent local and systemic pro-inflammatory responses.

**Results:**

*In vitro* assays demonstrated that the combination treatment achieved ∼80% cell killing, markedly inhibited cell migration, and significantly upregulated TNF-α and IL-12 secretion. *In vivo* evaluation using the 4T1 murine breast cancer model confirmed that this nanoplatform significantly suppressed primary tumor growth and pulmonary metastasis, with no appreciable systemic toxicity.

**Discussion:**

Collectively, this interfacial engineering strategy underscores the promising translational potential of Pickering emulsion-based nanoplatforms for aggressive solid tumor therapy.

## 1 Introduction

Breast cancer is the most commonly diagnosed malignant tumor in women worldwide (Xiong et al., 2025; Derakhshan and Reis-Filho, 2022; Wu et al., 2025). Triple-negative breast cancer (TNBC) is particularly difficult to treat, owing to its high tumor heterogeneity and the absence of hormone receptor expression. Standard clinical regimens relying on surgical resection, radiotherapy and conventional chemotherapy fail to achieve long-term disease control in most TNBC patients, who are prone to rapid tumor recurrence and distant metastasis (Zahra et al., 2025; Bianchini et al., 2021; Zhang et al., 2022). In recent years, cancer immunotherapy represented by immunogenic cell death (ICD) has brought new breakthroughs to tumor treatment (Lu et al., 2025; Jain et al., 2020; Gao et al., 2020). ICD induces the secretion of damage-associated molecular patterns (DAMPs) as well as tumor-associated antigens (TAAs); this cascade subsequently stimulates the organism’s immune response to identify and eliminate malignant tumor cells (Eteshola et al., 2021). Nevertheless, the immunosuppressive tumor microenvironment (TME) and low immunogenicity of TNBC greatly diminish the therapeutic efficacy of single-modality treatments (Guo et al., 2024; Nascimento and Ferreira, 2021). Thus, multimodal strategies that eradicate primary tumors while eliciting systemic antitumor immunity are urgently needed.

Combining photothermal therapy (PTT) with ferroptosis offers a viable strategy to address these therapeutic limitations (Lv et al., 2021; Yan et al., 2025; Wu et al., 2024; Cheng et al., 2024; Wang Y. et al., 2025). Ferroptosis represents a distinct subtype of regulated cell death, whose onset relies on iron-catalyzed peroxidation of cellular lipids (Li et al., 2020). PTT employs near-infrared (NIR) absorbing agents to generate local hyperthermia, which directly induces tumor cell thermal necrosis and acts as a powerful physical stimulus to trigger ICD (Ning et al., 2025). The combination of PTT and ferroptosis establishes a mutually reinforcing synergistic loop: local hyperthermia accelerates intracellular iron metabolism and promotes glutathione (GSH) depletion, ultimately leading to massive accumulation of lipid peroxides. Recent advances have broadened this paradigm via the development of diverse nanoplatforms. Representative examples include CuFeTe_2_ nanosheets that potentiate ferroptosis through photothermal enhancement, and plasmonic Au-Pd heterostructures that boost photoimmunotherapy by triggering ICD (Liu M. et al., 2025; Feng et al., 2023). In parallel, rational nanomedicine engineering to amplify ICD has emerged as a core strategy to optimize the therapeutic efficacy of cancer immunotherapy (Gao et al., 2020). Moreover, the introduction of imiquimod (R837), a classic TLR7 agonist, can effectively modulate macrophage polarization and boost pro-inflammatory cytokine secretion and enhance the infiltration of cytotoxic T lymphocytes (Adams et al., 2025; Graham-Gurysh et al., 2025; Gutmann et al., 2026). Nevertheless, clinical translation of TLR agonists faces major obstacles including fast systemic clearance and insufficient tumor accumulation. The Pickering emulsion platform mitigates such drawbacks by encapsulating R837 in the oil core. This strategy protects R837 against premature degradation and permits sustained, TME-triggered drug release within tumor tissue (Cao et al., 2024). As a result, local immunostimulatory activity is enhanced, whereas off-target systemic adverse effects are reduced. The integration of PTT-mediated tumor ablation, iron-dependent ferroptosis and R837-based immune activation aims to achieve both local tumor eradication and systemic anti-metastatic immune protection (Yazdimamaghani et al., 2025; Li et al., 2026). Precise implementation of this multimodal synergistic therapy relies on the design of stable and efficient delivery systems (Paredes-Toledo et al., 2025; Liu Y. et al., 2025). However, conventional polymeric nanoparticles and liposomes generally exhibit limited loading capacity for hydrophobic drugs. For liposomal systems, reported encapsulation efficiencies (EE%) typically range from approximately 53%–88%, while PLGA-based carriers yield EE values of 33%–80%. In comparison, Pickering emulsions achieve considerably higher encapsulation performance for hydrophobic drugs, with EE% consistently above 80% and frequently approaching 99% (Geng et al., 2024; Hu et al., 2022; Zhang et al., 2025; Ramos et al., 2024; Alhazzaa et al., 2025). Benefiting from this outstanding loading capability, together with irreversible particle adsorption that sustains long-term formulation stability and avoids the cytotoxicity induced by conventional surfactants, Pickering emulsions serve as a promising and robust platform for multimodal cancer therapy (Shukla et al., 2025).

Pickering emulsions (PEs) are stabilized by solid nanoparticles at the oil-water interface, and exhibit superior thermodynamic stability, higher drug loading capacity and superior biocompatibility compared with traditional surfactant-stabilized emulsions (Cui et al., 2024; Zia et al., 2023; Rayees et al., 2024; Ming et al., 2023; Li et al., 2025). More importantly, irreversible adsorption of nanoparticles at the interface forms a highly stable physical barrier that suppresses droplet coalescence and premature leakage of encapsulated cargoes-an enduring limitation of conventional liposomes and polymeric nanoparticles (Cao et al., 2024). This distinctive interfacial structure further supports compartmentalized designs, in which distinct therapeutic functions are spatially separated while exerting coordinated spatiotemporal effects. In this study, soybean oil was selected as the oil phase to encapsulate hydrophobic R837, facilitating the localized deliveryof R837 to tumor tissues. Iron-doped polydopamine (Fe-PDA) nanoparticles were synthesized and used as stabilizers for PE construction. Polydopamine possesses favorable NIR absorption capability and good biocompatibility as well as inherent biomimetic adhesion properties (Mrówczyński, 2018; Zhou et al., 2025; Li et al., 2023). After iron ion doping, the obtained Fe-PDA nanoparticles serve dual functions: they anchor at the oil-water interface as particulate stabilizers to maintain emulsion structural integrity, and simultaneously act as a sustainable iron source to trigger ferroptosis in tumor cells (Song et al., 2024; Zheng and Guan, 2023; Nie et al., 2025; Liu D. et al., 2025). Accordingly, Fe-PDA nanoparticles achieve multiple functions including interfacial stabilization, photothermal transduction, and iron release, combining these capacities in one unified nanoplatform. Such a compartmentalized design enables the spatiotemporally coordinated action of photothermal therapy, ferroptosis induction, and immune activation within the TME.

Despite these advantages, current Fe-PDA-based platforms are predominantly designed for monotherapy or simple bimodal combinations, and few studies have exploited Fe-PDA nanoparticles as Pickering stabilizers to construct a compartmentalized system that integrates photothermal ablation, ferroptosis induction and immune activation. Meanwhile, the low encapsulation efficiency and premature systemic leakage of hydrophobic adjuvants (R837) remain a long-standing bottleneck, which severely attenuates the synergistic efficacy of combined immunotherapy. To tackle these obstacles, we developed a multifunctional Pickering emulsion (Fe-PDA-PE-R837) and explored its application in triple-modal synergistic therapy against 4T1 breast cancer (Wang X. et al., 2025). Specifically, the interfacial Fe-PDA nanoparticles serve as photothermal agents for local tumor ablation, while providing iron sources to initiate ferroptosis. The inner soybean oil phase stably encapsulates R837 and achieves TME-responsive drug release, thereby triggering significant systemic immune responses. We systematically characterized the physicochemical properties, structural stability and *in vitro* drug release behavior of Fe-PDA-PE-R837. Using a 4T1 tumor-bearing mouse model, we systematically evaluated its synergistic anti-tumor efficacy, immune activation effect, and systemic biosafety through a series of *in vitro* and *in vivo* assays. Overall, this work highlights a facile interfacial engineering strategy with translational promise and underscores the value of integrating interfacial engineering with multiple cell death pathways for the treatment of aggressive breast cancer.

## 2 Materials and methods

### 2.1 Materials

Dopamine hydrochloride (DA, 98%) and Tris(hydroxymethyl) aminomethane (Tris, 99%) were purchased from Sigma-Aldrich. Ferric chloride hexahydrate (FeCl_3_·6H_2_O, analytical grade) was supplied by Sinopharm Chemical Reagent Co., Ltd.; imiquimod (R837, 98%) was provided by MedChemExpress (MCE); RPMI-1640 medium, high-glucose DMEM, fetal bovine serum (FBS), and trypsin were sourced from Gibco; Cell Counting Kit-8 (CCK-8) and Calcein-AM/PI Live/Dead Cell Staining Kit were supplied by Beyotime Biotechnology. The levels of TNF-α, IL-12, IFN-γ, Granzyme B, and TGF-β1 were measured using commercial ELISA kits obtained from Beyotime Biotechnology (Shanghai, China; Cat# PT512 for TNF-α, PI530 for IL-12, PI508 for IFN-γ) and Enzyme-linked Biotechnology (Shanghai, China; Cat# ml002067M for Granzyme B, ml002115M for TGF-β1).

### 2.2 Preparation and characterization of Fe-PDA NPs

Fe-PDA NPs were synthesized via a pre-doping strategy according to a previously reported method (Wang et al., 2021). In a typical procedure, 45 mg of dopamine hydrochloride and 15 mg of FeCl_3_·6H_2_O were dissolved in 130 mL of DI water and stirred at room temperature for 1 h. 20 mL of 100 mM Tris buffer (pH 9.0) was quickly added. The mixture immediately turned red and gradually transitioned to black over 0.5 h. After reacting for 12 h, the product was isolated by centrifugation, washed three times with DI water, and vacuum freeze-dried to yield the Fe-PDA NP powder.

Field-emission scanning electron microscopy (Hitachi S4800, FE-SEM) was adopted to characterize the morphological features and microstructural information of Fe-PDA nanoparticles. Dynamic light scattering (Malvern Nano-ZS, DLS) was utilized to detect hydrodynamic particle sizes and zeta potential values. The optical absorption properties were recorded using a UV-Vis-NIR spectrophotometer (200–1,000 nm, Shimadzu UV-3600). For photothermal performance evaluation, Fe-PDA dispersions at various concentrations (0.1–1 mg/mL) were exposed to an 808 nm laser at 1 W/cm^2^. Temperature changes were monitored in real time using an infrared thermal imaging camera, and the photothermal conversion efficiency was calculated via the classical cooling curve method. Water contact angles were measured with a goniometer on freeze-dried compressed Fe-PDA pellets, with equilibrium profiles recorded (Kruss GmbH FM4000).

### 2.3 Preparation and characterization of Fe-PDA-PE-R837

#### 2.3.1 Preparation of Fe-PDA-PE-R837

Pickering emulsions were prepared using a high-pressure homogenization method. Specifically, the oil phase was prepared by dissolving 0.8 mg of R837 in 4 mL of soybean oil via ultrasonication. For the aqueous phase, 120 mg of Fe-PDA nanoparticles were dispersed in 16 mL of deionized (DI) water and subjected to ultrasonication for 30 min. Subsequently, the oil and water phase were then mixed at a 2:8 volume ratio and homogenized via high-speed shearing at 15,000 rpm for 5 min to yield a coarse emulsion. The resulting mixture was further processed using high-pressure homogenization (80 MPa, 3 cycles) to obtain the Fe-PDA-PE-R837. For control experiments, blank Fe-PDA PE (without R837 loading) and PDA-PE-R837 (stabilized by pristine PDA nanoparticles) were prepared using the same procedure. To optimize R837 loading, the drug was dissolved in soybean oil at gradient concentrations (0, 0.1, 0.2, 0.3 mg/mL) prior to emulsions preparation. Additionally, emulsions with oil-to-water volume ratios of 1:9, 2:8, 3:7, 4:6, and 5:5 were formulated to assess the phase ratio effect on stability.

#### 2.3.2 Characterization of Fe-PDA-PE-R837

Morphology and Droplet Size. The micromorphology and droplet distribution of Fe-PDA-PE-R837 were initially observed under optical microscopy. Average droplet size and polydispersity index (PDI) were determined by DLS after appropriate dilution.

Stability Assessment. Physical stability was assessed through static storage and centrifugation assays. For storage stability, emulsions were kept at room temperature for 7 days, during which creaming, sedimentation, or phase separation was closely monitored. Mechanical stability was assessed by centrifuging the emulsion samples at 5,000 rpm for 10 min to check for droplet coalescence and oil-water separation. Rheological measurements were conducted on a TA Instruments DHR-2 rheometer with a 40 mm parallel-plate geometry. Frequency sweeps were performed from 0.01 to 10 Hz at a constant strain of 1% (within the linear viscoelastic region) at 25 °C. Fresh (day 0) and 7-day-stored emulsions were tested in parallel.

Drug Loading and *In Vitro* Release. The loading capacity (LC) and encapsulation efficiency (EE) of R837 in the Pickering emulsions were determined using a dialysis method. R837 concentration was quantified by UV-Vis spectrophotometry at 248 nm against a standard calibration curve (Supplementary Figure S1). For release studies, Fe-PDA-PE-R837 were sealed in dialysis bags (MWCO 3500) and immersed in four different media to mimic physiological and tumor microenvironments: (1) pH 7.4 PBS with 0.5% (v/v) Tween 80; (2) pH 5.0 acetate-buffered saline (ABS) with 0.5% Tween 80 (Wu et al., 2025); pH 5.0 ABS with 0.5% Tween 80 and 10 mM H_2_O_2_; and (4) pH 5.0 ABS with 0.5% Tween 80 and 10 mM glutathione (GSH). At preset intervals, aliquots were withdrawn and replaced with fresh media to maintain sink conditions. Cumulative release profiles of R837 and Fe were plotted against incubation time.

Photothermal Performance. The photothermal properties of the Fe-PDA-PE-R837 were investigated under 808 nm laser irradiation. Samples in quartz cuvettes were irradiated with an 808 nm laser at 1 W/cm^2^, and temperature fluctuations were recorded by an infrared thermal camera. Aqueous Fe-PDA dispersions at equivalent Fe concentrations were tested in parallel for comparison.

*In vitro* hemolysis assay. Hemocompatibility was assessed using fresh mouse red blood cells (RBCs). Briefly, 2% (v/v) RBC suspensions were incubated with serial concentrations of Fe-PDA-PE-R837 (0.5, 1.0, 1.5 and 2.0 mg/mL Fe equivalent) at 37 °C for 3 h. DI water served as positive controls. After centrifugation at 3,000 rpm for 10 min, the absorbance of the supernatant at 540 nm was measured, and the hemolysis ratio was calculated.

### 2.4 Cell culture and *in vitro* grouping

Mouse breast carcinoma 4T1 cell line and RAW 264.7 murine macrophage cell strain were purchased from the Cell Bank affiliated with the Chinese Academy of Sciences in Shanghai. RPMI-1640 complete medium served as the culture system for 4T1 cells, whereas high-glucose DMEM was applied for the cultivation of RAW 264.7 macrophages. Both media were supplemented with 10% FBS and 1% penicillin-streptomycin. All cell lines were incubated at a 37 °C with 5% CO_2_ (CLM-170B-8-NF, ESCO).

Seven experimental groups were designed to explore the *in vitro* therapeutic mechanisms: Group 1 (Control): untreated cells; Group 2 (Fe-PDA): cells treated with pristine Fe-PDA nanoparticles; Group 3 (Fe-PDA-PE): cells treated with blank Pickering emulsions; Group 4 (Fe-PDA-PE-R837): cells treated with R837-loaded emulsions without laser; Group 5 (Fe-PDA-PE + Laser): cells treated with blank emulsions followed by 808 nm laser irradiation (0.6 W/cm^2^, 5 min); Group 6 (Fe-PDA-PE-R837+Laser): cells treated with the full formulation followed by 808 nm laser irradiation (0.6 W/cm^2^, 5 min); Group 7 (Fe-PDA-PE-R837+Laser + Fer-1): cells pre-incubated with 5 μM Fer-1 for 1 h before the full treatment and laser exposure.

### 2.5 *In vitro* cytotoxicity assay

The CCK-8 colorimetric test was utilized to evaluate the inhibitory cytotoxic effect on 4T1 tumor cells. Cells were plated onto 96-well culture plates with a seeding density of 1 × 10^4^ cells per well and incubated overnight to achieve full attachment. All iron-loaded nanotherapeutic groups were treated with identical iron levels corresponding to 50 μg/mL Fe. For Groups 5 to 7, after 4 h of co-cultivation with the preparations, 808 nm near-infrared light (power density: 0.6 W/cm^2^) was applied for a 5-min irradiation period, and the cells were continuously cultured for another 24 h afterwards. Afterwards, 10 μL CCK-8 reagent was supplemented into every single well. Following a 2-h incubation interval, a microplate spectrophotometer was employed to measure optical density values at 450 nm, and these readings were further calculated to quantify cellular survival rates.

In parallel, live/dead staining with Calcein-AM/PI was performed. After 24 h of treatment, cells were rinsed twice with PBS and incubated with a staining cocktail (Calcein-AM for live cells, green; PI for dead cells, red) at 37 °C in the dark for 15 min, then immediately imaged under a Nikon Ti2 confocal microscope.

### 2.6 Intracellular iron content analysis via ICP-MS

Cellular uptake of iron-containing formulations was quantified by inductively coupled plasma mass spectrometry (ICP-MS). 4T1 cells (2 ✕ 10^5^ per well) were cultured in 6-well plates and incubated with the iron-containing formulations for 4 h. Cells were then washed three times with cold PBS, trypsinized, counted, and pelleted. The pellets were digested with 500 μL of 65% high-purity nitric acid (HNO_3_) at 90 °C for 2 h until clear. Digested solutions were diluted to 10 mL with DI water, and Fe content was measured on an Agilent ICP-MS system against a linear external standard curve (Supplementary Figure S2).

For soluble labile iron (SLI) quantification, cell lysates or tumor homogenates were transferred to a 3 kDa ultrafiltration tube and centrifuged at 12,000 × g for 30 min at 4 °C. The filtrate containing free iron was collected, digested with 65% HNO_3_, and quantified by ICP-MS following the same procedure. All iron data were normalized to cell number or tissue mass.

### 2.7 Cell wound healing assay

The anti-migratory effects of the treatments were evaluated by wound healing assay. 4T1 cells were cultured in 6-well plates to confluence. A linear scratch was created across the cell monolayer with a sterile 200 μL pipette tip. Debris was removed by gentle PBS washing, and cells were incubated with serum-free medium containing the respective treatments (Groups 1–7). Images of the scratched areas were captured at 0 and 24 h post-scratching. Wound closure area was measured with ImageJ software to calculate the healing rate.

### 2.8 Intracellular MDA and GSH assays

Ferroptosis induction was verified by quantifying intracellular malondialdehyde (MDA, a lipid peroxidation marker) and reduced glutathione (GSH) levels. After 24 h of treatment, 4T1 cells were harvested and lysed using the specialized buffer provided in the assay kits. Lysates were centrifuged to collect supernatant. MDA (via the thiobarbituric acid method) and GSH contents were determined strictly per the manufacturers’ protocols. All data were normalized to total protein concentration determined via a BCA protein assay kit.

### 2.9 Macrophage stimulation via conditioned medium

A conditioned medium (CM) model was established to evaluate the immunostimulatory effect of soluble factors released from dying tumor cells on macrophages. After 24 h of the indicated treatments, 4T1 culture supernatants were collected and centrifuged at 2,000 rpm and 4 °C for 10 min to remove debris and non-internalized particles. The clear supernatants were collected as CM for each group.

RAW 264.7 macrophages were seeded in 24-well plates at a density of 5 ✕ 10^4^ cells per well. After adhesion, the medium was replaced with a 1:1 (v/v) mixture of CM and fresh complete medium. Following 24 h of co-incubation, macrophage culture supernatants were harvested, and pro-inflammatory cytokine (TNF-α and IL-12) secretion was quantified using ELISA kits according to the manufacturers’ instructions.

### 2.10 *In vivo* tumor model construction and treatment protocols

#### 2.10.1 Establishments of the *in vivo* breast cancer model

Female specific-pathogen-free (SPF) BALB/c mice aged 6 weeks and weighing approximately 20 g were obtained from Changzhou Cavens Laboratory Animal Co., Ltd. All *in vivo* experimental operations were sanctioned by the Animal Ethics Committee of Changzhou University (Ethics Approval Number: 20240328001). The entire animal study was implemented in accordance with the ARRIVE guidelines and strictly abided by local laboratory animal welfare and management regulations. The humane endpoints were defined as tumor volume exceeding 2000 mm^3^, tumor ulceration, or >20% body weight loss, in which case the mice would be euthanized prematurely. In practice, all mice were euthanized when the mean tumor volume of the control group reached approximately 600 mm^3^ (day 18 post-treatment initiation), as further tumor growth would have led to spontaneous intratumoral necrosis, potentially interfering with the detection of ferroptosis-related molecular markers and histological assessments. For intratumoral injection procedures, mice were anesthetized with pentobarbital sodium at a dose of 40 mg/kg via intraperitoneal injection. At the experimental endpoint, all mice were euthanized by cervical dislocation performed by trained personnel, without the use of any chemical euthanizing agents. 4T1 tumor cells in the logarithmic growth stage were collected and resuspended in sterile normal saline to prepare a uniform cell suspension with a concentration of 1 × 10^7^ cells/mL. Following a one-week adaptive feeding period, each mouse received a subcutaneous injection of 100 μL cell suspension (containing 1 × 10^6^ cells) at the right flank site. Tumor growth was monitored daily by measuring the major longitudinal diameter (a) and transverse diameter (b) with a digital caliper. Tumor volume (V) was calculated as: V = a × b^2^/2.

#### 2.10.2 *In vivo* antitumor evaluation

When tumors reached 100–150 mm^3^, mice were randomly assigned to five cohorts (n = 6 per group). Intratumoral injections of the respective formulations (100 μL per mouse) were administered twice weekly over a period of 3 weeks (with all mice euthanized on day 18 post-treatment initiation when the mean tumor volume of the control group reached approximately 600 mm^3^). Ferrostatin-1 was dissolved in DMSO/saline (5%, v/v) to 0.5 mg/mL and injected intravenously at 5 mg/kg 1 h before laser irradiation. For photothermal groups, tumor sites were irradiated with an 808 nm laser (1.5 W/cm^2^, 5 min) at 4 h post-injection. A higher power density (1.5 W/cm^2^) was used *in vivo* relative to *in vitro* settings to compensate for tissue attenuation.

The cohorts were: (1) Control: normal saline; (2) Fe-PDA-PE: blank Fe-PDA-PE emulsions (5 mg/kg Fe equivalent); (3) Fe-PDA-PE + Laser: blank emulsions plus 808 nm laser; (4) Fe-PDA-PE-R837+Laser + Fer-1: R837-loaded emulsions (5 mg/kg Fe; 0.5 mg/kg R837) plus laser, with Fer-1 pretreatment; (5) Fe-PDA-PE-R837+Laser: complete formulation (5 mg/kg Fe; 0.5 mg/kg R837) plus laser.

### 2.11 RT-qPCR analysis

The transcriptional expression levels of ferroptosis-related genes (*Gpx4, Slc7a11, Ptgs2, Acsl4,* and *Fth1*) were detected via real-time quantitative polymerase chain reaction (RT-qPCR). Approximately 50 mg of isolated 4T1 tumor tissues were homogenized in 1 mL TRIzol Reagent to extract total cellular RNA. The concentration and purity of isolated RNA specimens were determined based on the absorbance value at 260 nm, and all qualified samples were standardized to a consistent concentration of 0.5 μg/μL. After genomic DNA removal treatment, 2.0 μg of total RNA was reverse-transcribed into complementary DNA (cDNA) using the FastKing RT Kit equipped with gDNAse. Subsequent quantitative PCR amplification was carried out on a real-time PCR detection system with SYBR Premix Ex TaqTM (Takara) as the fluorescent dye reagent. The relative expression levels of target genes were quantified utilizing the 2^−ΔΔCT^ comparative threshold cycle method. Each experimental reaction was performed in biological triplicate to ensure data reliability. Primer sequences (*GAPDH*, *Gpx4*, *Slc7a11*, *Ptgs2, Acsl4*, and *Fth1*) are listed in Table 1 and were synthesized by Shanghai Shenggong Bioengineering Technology Service Co., Ltd.

**TABLE 1:** Primers for RT-qPCR in this study.

Gene	Sense primer	Antisense primer
GAPDH	AGG​TCG​GTG​TGA​ACG​GAT​TTG	TGT​AGA​CCA​TGT​AGT​TGA​GGT​CA
*Gpx4*	GCC​TCG​GTT​TAC​GAA​TCA​GG	CTC​ATG​AAG​CAT​CAA​CAC​CTG​G
*Slc7a11*	CCA​GTT​ACA​CAT​ACA​TCC​CCG	CTT​CTC​CAC​AGG​CGA​TTG​AC
*Ptgs2*	CTG​GTG​AAG​CTG​TCC​AAG​AC	TGC​AGC​CAT​TTC​CTT​CTC​TC
*Acsl4*	CAC​CAT​GTT​TGC​TGG​TAG​CC	GGC​AGT​CAC​ACC​ACG​TAG​AT
*Fth1*	CGT​GGA​AGA​GTG​CCG​TGT​T	GGG​CTT​CAC​TCA​CTG​TCC​AC

### 2.12 ELISA analysis

Systemic and local immune responses were evaluated by measuring TNF-α, IL-12, IFN-γ, Granzyme B, and TGF-β1 concentrations in mouse serum and tumor tissues via ELISA. All kit components were equilibrated to room temperature for 20 min before use. Standard curves were fitted with a four-parameter logistic (4PL) regression model on a log-log scale. All calibration curves showed good linear fitting (Supplementary Figures S3-S7). Briefly, 100 μL of sample or standard was added to each well and incubated at 37 °C for 2 h. After removing the residual solution and conducting five repeated rinses with washing buffer, 100 μL of biotin-labeled detection antibody was supplemented to each well, followed by a 60-min incubation period at room temperature. After a further round of washing, 100 μL of HRP-streptavidin conjugate was added, and the culture plate was incubated in a dark environment for 20 min. The enzymatic reaction was terminated by adding 50 μL of stop buffer. Immediately afterward, the optical density at 450 nm was recorded using a microplate reader. The concentrations of target cytokines were finally calculated according to the established standard calibration curves.

### 2.13 Statistical analysis

Statistical analysis for pairwise comparisons was performed using the Student’s t-test. For multi-group comparisons, one-way ANOVA was performed followed by Tukey’s post-hoc test for pairwise comparisons among all groups. Normality was assessed using the Shapiro–Wilk test, and homogeneity of variances was verified by Levene’s test prior to ANOVA. All quantitative image analyses (wound closure measurement, live/dead cell counting) were performed blindly by two independent investigators. All experimental data in this study were expressed as mean ± standard deviation (SD), which were derived from no fewer than three independent *in vitro* experiments. All statistical analyses were implemented with GraphPad Prism 8 software. A *P*-value of less than 0.05 was defined as the threshold for statistically significant differences in all analyses.

## 3 Results and discussion

### 3.1 Synthesis and characterization of Fe-PDA nanoparticles

As a Pickering emulsion stabilizer and dual-modal therapeutic agent (photothermal therapy and ferroptosis), we first synthesized PDA and Fe-PDA nanoparticles. As shown in Figures 1A,B, PDA was prepared via dopamine self-polymerization using NH_4_OH, while Fe-PDA was synthesized by coordinating dopamine with Fe^3+^ followed by polymerization in an alkaline Tris buffer (pH = 9). DLS measurements gave average hydrodynamic diameters of 143.3 ± 4.2 nm and 163.7 ± 7.5 nm for PDA and Fe-PDA, respectively (Figures 1C,D; Supplementary Table S1). The zeta potentials of PDA and Fe-PDA nanoparticles were determined to be −30.0 ± 1.4 mV and −20.9 ± 1.8 mV, respectively, indicating favorable colloidal stability (Supplementary Figure S8). The modest size increase after iron doping supports the successful incorporation of Fe^3+^ during polymerization. SEM images (Figures 1E,F) confirmed uniform spherical morphologies and good dispersibility for both nanoparticles-traits that favor their use as solid Pickering stabilizers.

**FIGURE 1:**
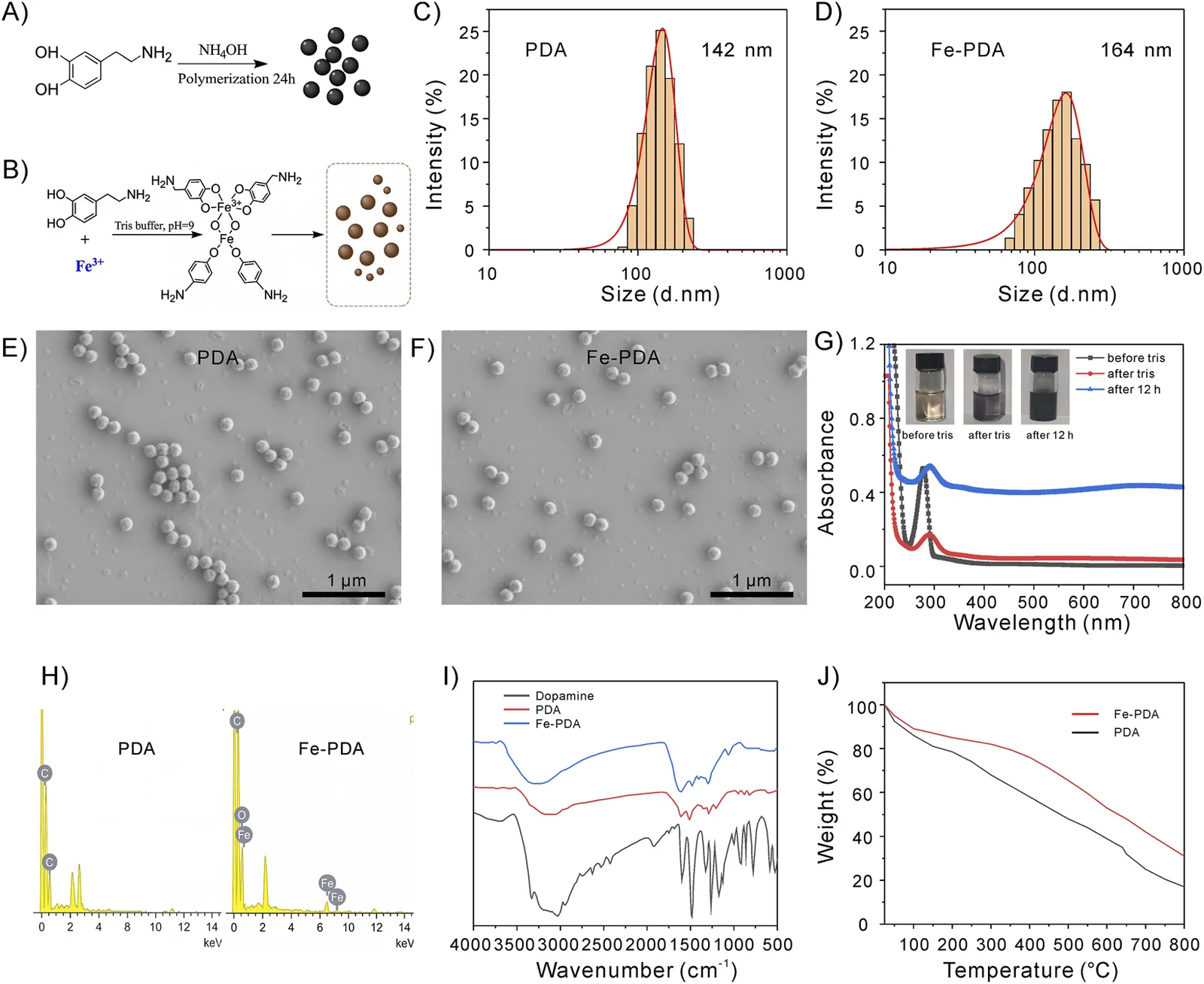
Synthesis and characterization of PDA and Fe-PDA nanoparticles. **(A,B)** Schematic il-lustrations of the synthesis of **(A)** polydopamine (PDA) and **(B)** Fe-doped PDA (Fe-PDA) nano-particles. **(C,D)** DLS size distributions and **(E,F)** SEM images of PDA and Fe-PDA nanoparticles, respectively. Data represent a typical measurement from three independent experiments (n = 3). **(G)** UV-vis absorption spectra at different stages of Fe-PDA synthesis (inset: digital photographs of the corresponding solutions). **(H)** Energy-dispersive X-ray (EDX) spectra of PDA and Fe-PDA. **(I)** FTIR spectra of dopamine, PDA, and Fe-PDA. **(J)** TGA curves of PDA and Fe-PDA.

UV-vis spectroscopy was employed to monitor the synthesis process of Fe-PDA in real time (Figure 1G). After the addition of Tris buffer followed by a 12 h reaction, the solution turned deep black (inset of Figure 1G) and exhibited an intense, featureless absorption band extending across the UV to NIR region. This broad absorption profile is a typical characteristic of polydopamine and lays the material foundation for subsequent PTT.

To confirm successful iron doping, energy-dispersive X-ray (EDX) spectroscopy was conducted (Figure 1H). Distinct Fe peaks were detected in the Fe-PDA sample, whereas no corresponding signals appeared in pristine PDA. Furthermore, Fourier transform infrared (FTIR) spectroscopy (Figure 1I) showed shifts in characteristic peaks relative to those of pure dopamine and pristine PDA, corroborating the formation of metal coordination bonds between catechol groups and Fe^3+^ ions. Finally, thermogravimetric analysis (TGA, Figure 1J) revealed that Fe-PDA retained a higher residual mass (∼30%) than pristine PDA (∼15%) at 800 °C, which is mainly ascribed to the formation of inorganic iron-containing residues after high-temperature carbonization (Zhu et al., 2022). Water contact angle measurements verified the favorable wettability of Fe-PDA, which supports its ability to stabilize O/W emulsions (Supplementary Figure S9). Collectively, these compositional and structural analyses verify the successful fabrication of Fe-PDA nanoparticles.

### 3.2 Preparation, stability, and responsive release behavior of pickering emulsions

As shown in Figure 2A, the Fe-PDA-stabilized Pickering emulsion adopted a typical oil-in-water (O/W) structure. The intrinsic surface properties of Fe-PDA nanoparticles promoted their adsorption at the oil-water interface, with a water contact angle of θ_w_ < 90°, a condition that thermodynamically favors the formation of O/W emulsions (Xia et al., 2025). This interfacial assembly was further validated by confocal laser scanning microscopy (CLSM, Figure 2B). Nile Red-labeled oil droplets (red fluorescence) were uniformly dispersed in the FITC-stained continuous aqueous phase (green fluorescence), and a distinct non-fluorescent boundary was observed around each oil core. This boundary corresponds to the densely packed Fe-PDA nanoparticle layer, which acts as a physical barrier to prevent droplet coalescence and stabilize the emulsion system. Of note, the Fe-PDA nanoparticles themselves maintained excellent colloidal stability in both saline and serum media over 7 days (Supplementary Figures S10 and S11), which is a prerequisite for long-term emulsion stability.

**FIGURE 2:**
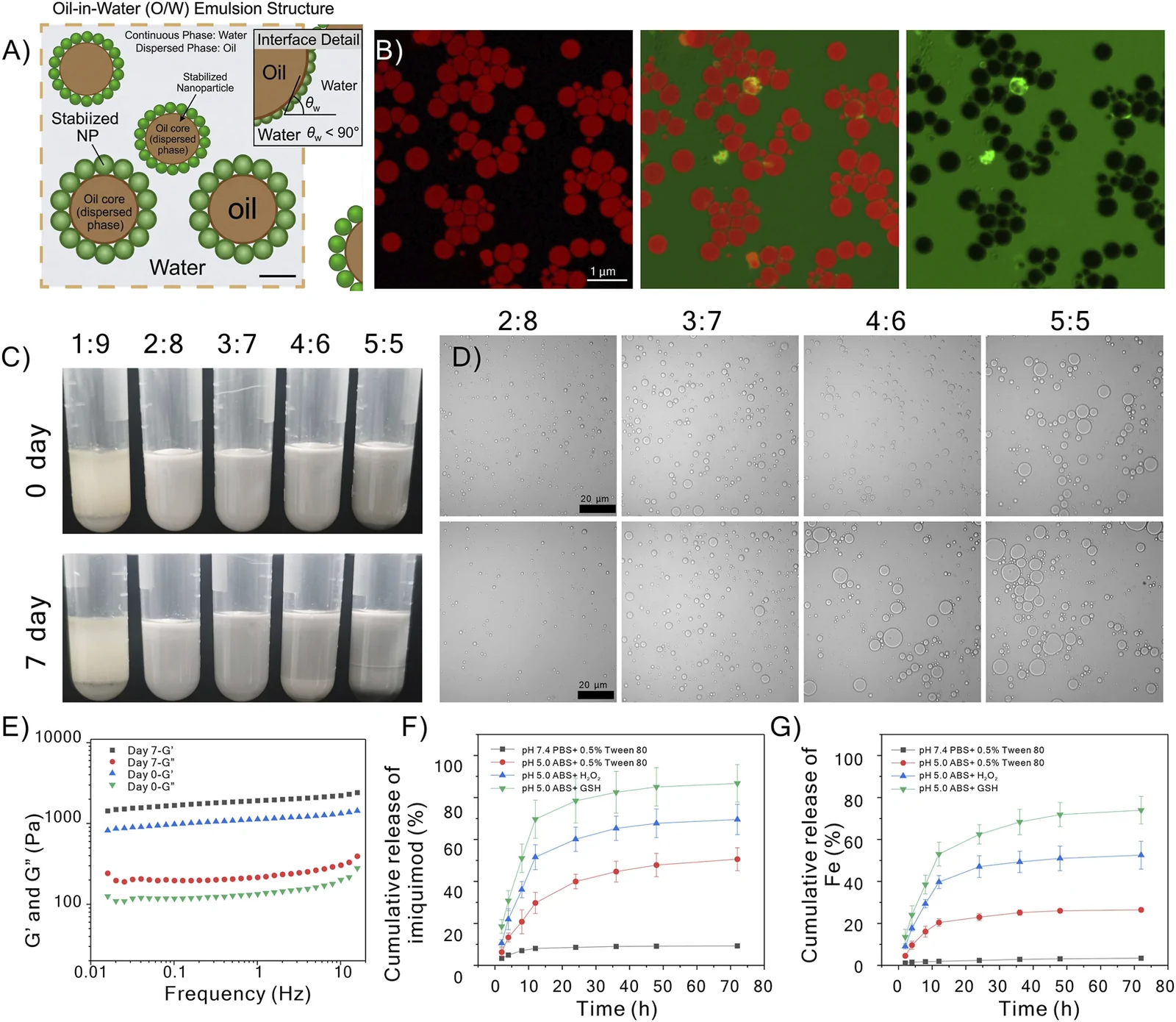
Characterization and *in vitro* release behavior of the Pickering emulsions. **(A)** Schematic of the O/W Pickering emulsion structure and interfacial configuration of stabilizing nanoparticles (θw < 90°). **(B)** CLSM images of the emulsions, with the oil and aqueous phases stained by 0.1 wt% Nile Red (red) and 0.1 wt% FITC (green), respectively. **(C)** Photographs and **(D)** corresponding optical microscopy images of the emulsions at varying oil-to-water ratios on day 0 and day 7. **(E)** Frequency-sweep rheological profiles of storage modulus (G′) and loss modulus (G″) for the fresh (day 0) and aged (day 7) emulsions. **(F,G)** Cumulative release profiles of **(F)** imiquimod and **(G)** Fe from the emulsions under different conditions.

The physical stability of emulsions prepared with oil-to-water volume ratios ranging from 1:9 to 5:5 was assessed over 7 days of static storage. Macroscopic observations (Figure 2C) showed that the emulsion with a 1:9 oil-to-water ratio underwent partial sedimentation, whereas formulations with higher oil fractions (2:8 to 5:5) retained a homogeneous milky appearance with no visible creaming or phase separation after 7 days. Consistently, optical microscopy images (Figure 2D) revealed that average droplet size increased gradually with rising oil content, but no extensive droplet coalescence was detected for formulations between 2:8 and 5:5 during storage. For the drug-loaded emulsion Fe-PDA-PE-R837, quantitative dynamic light scattering (DLS) monitoring further verified that its mean droplet size remained nearly unchanged within 5 days of storage (Supplementary Figure S12). Such high resistance to demulsification arises primarily from the steric hindrance conferred by the interfacial Fe-PDA nanoparticle layer.

Frequency-sweep rheological tests were conducted on fresh (day 0) and 7-day-stored emulsion samples to characterize the viscoelastic properties of the emulsion network (Figure 2E). Rheological measurements were performed in triplicate on independently prepared emulsion batches, and representative curves are shown. Over the entire tested frequency range, the storage modulus (G′) was consistently higher than the loss modulus (G″) for both samples, indicating a predominantly elastic, gel-like network structure in the emulsion system (Shang et al., 2019). Notably, both moduli showed a slight increase rather than a decrease after 7 days of storage, suggesting that the packing of interfacial nanoparticles remained largely intact and the network structure was well preserved over time (Mohammed et al., 2025).

Prior to release testing, the loading efficiency of R837 was optimized by adjusting the initial drug concentration, yielding a maximum encapsulation efficiency of ∼90% (Supplementary Figures S13 and S14). The *in vitro* release profiles of co-encapsulated imiquimod (R837) and iron were then investigated under conditions mimicking physiological and tumor microenvironments (Figures 2F,G). In neutral medium (pH 7.4 PBS containing 0.5% Tween 80), less than 20% of both cargoes was released over 72 h, reflecting excellent structural stability of the emulsion under normal physiological conditions and minimal premature leakage before reaching target sites. In contrast, release of both drug and iron was significantly accelerated in acidic buffer (pH 5.0 acetate buffer), and this effect was further enhanced by the addition of H_2_O_2_ or GSH. The fastest release was observed in the presence of both acidic pH and high GSH concentration, which can be attributed to the degradation of the polydopamine skeleton in an acidic reductive environment. This stimuli-responsive release behavior effectively reduces premature payload leakage in normal tissues while enabling rapid drug and ion release in the tumor microenvironment. Of note, all four release profiles reached a plateau within the 72-h observation period. The Fe cumulative releases at 72 h were 3.4% ± 0.5%, 26.4% ± 0.9%, 52.5% ± 6.6%, and 73.9% ± 6.5% for the pH 7.4 PBS, pH 5.0 ABS, pH 5.0 ABS + H_2_O_2_, and pH 5.0 ABS + GSH conditions, respectively, demonstrating a clear stimuli-responsive release pattern dependent on the acidic and reductive tumor microenvironment.

### 3.3 *In vitro* photothermal performance and hemocompatibility evaluation of Fe-PDA PE

First, the photothermal behavior of Fe-PDA PE was characterized under 808 nm laser irradiation. Thermal images (Figure 3A) and quantitative heating profiles (Figures 3B,C) showed that the PBS blank group generated negligible heat upon irradiation, ruling out thermal contributions from the solvent and laser itself. Fe-PDA PE exhibited rapid and tunable temperature elevation, which depended on both Fe-equivalent concentration and laser power density: at a fixed laser power density of 0.6 W/cm^2^, the plateau temperature increased steadily with rising particle dosage; when the Fe-equivalent concentration was held constant at 0.25 mg/mL, the equilibrium temperature rose as laser power density increased from 0.1 to 1.2 W/cm^2^. This tunable photothermal response enables precise modulation of treatment intensity for tumor photothermal ablation.

**FIGURE 3:**
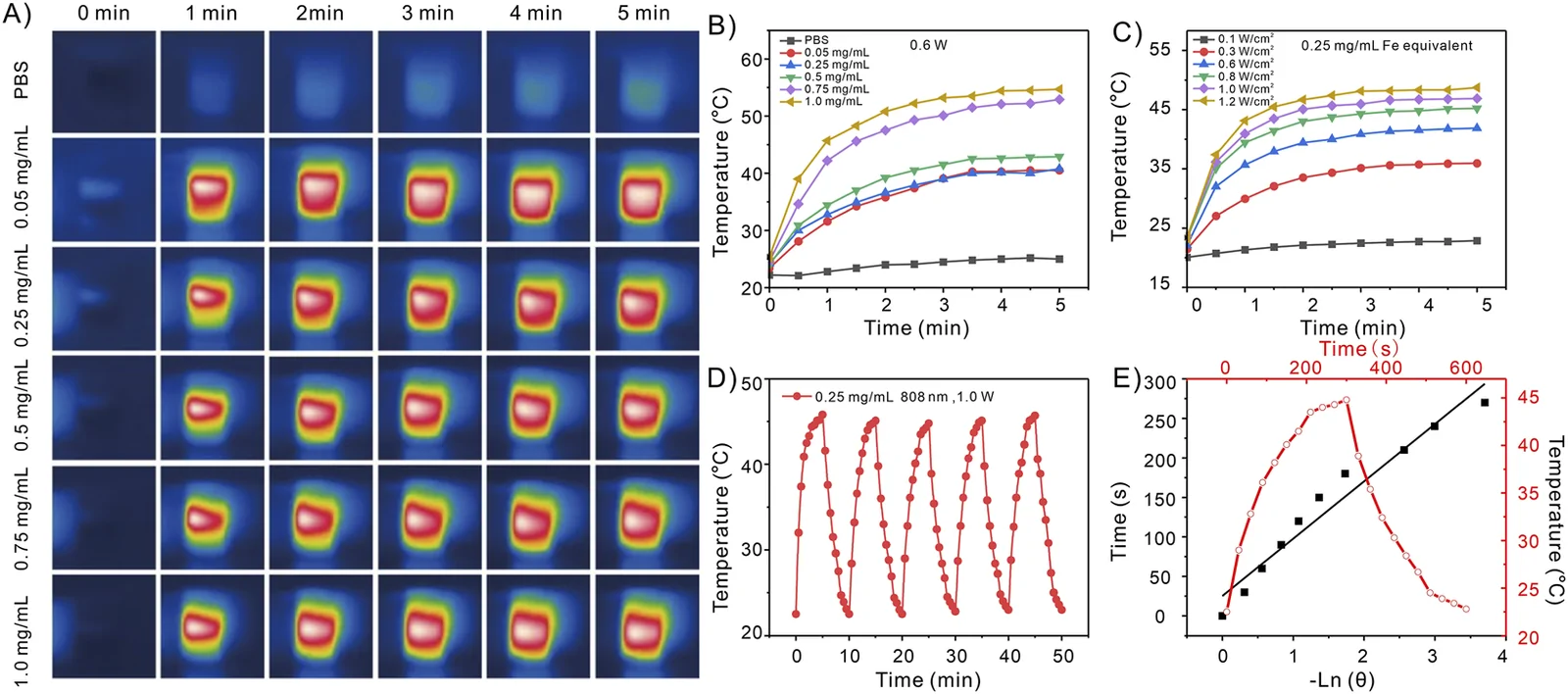
Characterization of photothermal performance. **(A)** Infrared thermal imaging snapshots of PBS and Fe-PDA PE with serial Fe equivalent concentrations (0.05–1.0 mg/mL) captured at 0–5 min upon continuous 808 nm laser irradiation. **(B)** Temperature elevation profiles of samples with varying Fe concentrations under 808 nm laser at power density of 0.6 W/cm^2^. **(C)** Heating curves of 0.25 mg/mL Fe-equivalent Fe-PDA PE exposed to 808 nm laser with gradient power densities ranging from 0.1 to 1.2 W/cm^2^. **(D)** Five repeated on-off laser irradiation cycles to test photothermal stability of 0.25 mg/mL Fe-PDA dispersion under 808 nm, 1.0 W laser. **(E)** Linear regression of -Ln(θ) against cooling time to determine photothermal conversion efficiency.

For reliable *in vivo* PTT applications, excellent photothermal stability and high photothermal conversion efficiency are critical prerequisites. Five intermittent on-off laser irradiation cycles (Figure 3D) yielded highly consistent peak temperatures across all runs, with no attenuation of photothermal activity after repeated 808 nm exposure, verifying the favorable photothermal stability of Fe-PDA PE. By linear fitting of the cooling curve against -lnθ (Figure 3E), the photothermal conversion efficiency of Fe-PDA PE was determined to be 38.5%. Together with its tunable heating capacity, excellent cycling stability and 38.5% light-to-heat conversion efficiency, Fe-PDA PE can serve as a robust photothermal agent for synergistic breast cancer therapy (Wang et al., 2021).

Beyond photothermal performance, hemocompatibility is an essential safety requirement for intravenously administered nanomedicines to avoid systemic adverse effects. Accordingly, a hemolysis assay using mouse erythrocytes was performed to assess the blood compatibility of Fe-PDA-PE-R837 (Supplementary Figures S15-S17), with DI water set as the positive controls. After 3 h of incubation at 37 °C, negligible hemoglobin leakage was detected across all tested concentrations (0.5, 1.0, 1.5, and 2.0 mg/mL Fe equivalent). Quantitatively, all hemolysis ratios were below 5%, which meets the international safety threshold for biomedical materials. Collectively, these results demonstrate the excellent hemocompatibility of the nanoplatform and support its feasibility for subsequent *in vivo* investigations.

### 3.4 *In vitro* synergistic antitumor efficacy and underlying mechanisms

We next conducted a series of *in vitro* cellular assays to systematically evaluate the antitumor efficacy of Fe-PDA-PE-R837 and its underlying synergistic mechanisms. As shown in Figures 4A,C, cells in the untreated control group and all non-irradiated treatment groups (Fe-PDA, Fe-PDA-PE, and Fe-PDA-PE-R837) showed predominant green fluorescence with cell viabilities above 90%, verifying the favorable biocompatibility of all formulations under dark conditions. Upon 808 nm laser irradiation, the Fe-PDA-PE + Laser group displayed strong red fluorescence (indicating dead cells) and a marked decline in cell viability to ∼20%, reflecting potent photothermal ablation efficacy. The incorporation of the R837 adjuvant (Fe-PDA-PE-R837 + Laser) further enhanced this cytotoxic effect. Critically, co-incubation with the ferroptosis inhibitor ferrostatin-1 (Fer-1) partially restored cell viability and attenuated dead-cell signals, confirming that ferroptosis is a significant contributor to the cell-killing effect, alongside PTT.

**FIGURE 4:**
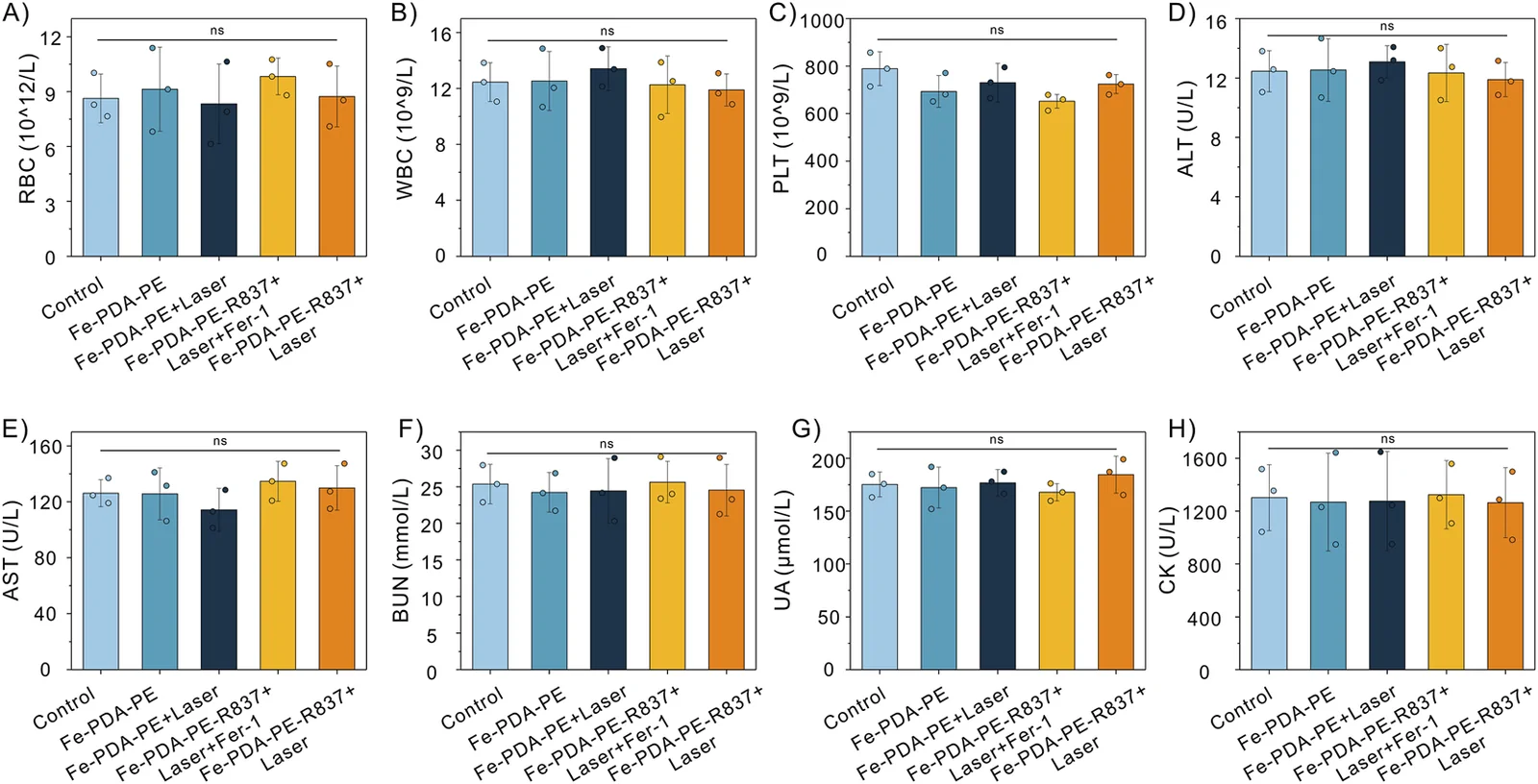
*In vitro* antitumor evaluation of the Fe-PDA-derived nanotherapeutics. **(A)** Fluorescent images of live/dead stained tumor cells after diverse treatment regimens (green fluorescence represents viable cells, while red fluorescence indicates dead cells). Scale bar: 100 μm. **(B)** Typical microscopic images obtained from the scratch wound healing assay for cell migration evaluation. Scale bar: 200 μm. **(C–H)** Quantitative analysis of **(C)** cell viability, **(D)** wound closure rate, **(E)** MDA content, **(F)** GSH content, and the secretion levels of pro-inflammatory cytokines **(G)** TNF-α and **(H)** IL-12.

The inhibitory effect of the formulations on tumor cell migration was evaluated via a scratch wound healing assay (Figures 4B,D). Cells in the control and non-irradiated groups exhibited rapid wound closure, whereas the combination treatment (Fe-PDA-PE-R837 + Laser) exerted the strongest suppression on cell migration. This anti-migratory effect was partially reversed by Fer-1 supplementation, corroborating that ferroptosis induction directly contributes to the inhibition of tumor cell motility.

To further elucidate the ferroptosis pathway, we quantified intracellular levels of malondialdehyde (MDA, a lipid peroxidation marker) and glutathione (GSH) (Figures 4E,F). All non-irradiated groups maintained redox homeostasis comparable to that of the control group. In contrast, the Fe-PDA-PE-R837 + Laser treatment induced severe GSH depletion and pronounced MDA accumulation, indicative of disrupted antioxidant defense and extensive lipid peroxidation (Deng et al., 2022). As expected, Fer-1 addition significantly replenished the intracellular GSH pool and reduced MDA production. These biochemical results support a mechanism whereby the photothermal effect of Fe-PDA accelerates intracellular iron release, triggering a lethal lipid peroxidation cascade driven by the Fenton reaction.

We next assessed cellular internalization and intracellular iron release of the nanoplatforms by measuring total cellular iron and soluble labile iron (SLI) in 4T1 cells via ICP-MS (Supplementary Figure S18). Cells treated with all Fe-containing formulations exhibited substantially higher total intracellular iron levels than control cells, confirming efficient cellular uptake of the nanoparticles. In the absence of laser irradiation, intracellular SLI levels remained low, suggesting that Fe ions were stably encapsulated within the polydopamine skeletons with minimal premature leakage. After 808 nm laser irradiation, SLI levels increased by more than twofold, whereas total iron content remained unchanged. This finding directly demonstrates that photothermal heating promotes degradation of the Fe-PDA framework and triggers massive release of catalytic free iron into the cytoplasm. Meanwhile, Fer-1 co-incubation had no significant effect on SLI concentrations, consistent with its mode of action: it scavenges downstream lipid peroxides rather than modulating upstream iron metabolism.

Finally, we measured the secretion of pro-inflammatory cytokines (TNF-α and IL-12) to evaluate the immunostimulatory capacity of the formulations (Figures 4G,H). Bare Fe-PDA nanoparticles induced negligible cytokine secretion. The R837-loaded formulation (Fe-PDA-PE-R837) moderately upregulated cytokine production, attributable to the intrinsic Toll-like receptor 7 (TLR7) agonistic activity of R837. Notably, laser irradiation further boosted cytokine secretion to the highest levels in the combination treatment group. This enhanced cytokine secretion, suggesting immune activation, can be explained by the fact that photothermal and ferroptosis-induced cellular damage releases tumor-associated antigens and damage-associated molecular patterns (DAMPs), which act synergistically with the R837 adjuvant. Collectively, these *in vitro* results demonstrate that the Fe-PDA-stabilized Pickering emulsion nanoplatform possesses triple-modal synergistic therapeutic potential, integrating PTT, ferroptosis, and immunotherapy.

### 3.5 *In vivo* synergistic antitumor efficacy

To assess the *in vivo* therapeutic efficacy of the Fe-PDA-PE, we established 4T1 breast tumor-bearing mouse models and administered different treatment regimens over an 18-day observation period. As shown by representative tumor photographs (Figure 5A) and terminal tumor weight quantification (Figure 5B), the untreated control and non-irradiated Fe-PDA-PE groups exhibited rapid, unrestrained tumor progression. In contrast, mice treated with Fe-PDA-PE plus 808 nm laser showed moderate tumor growth inhibition, which was mainly attributed to direct photothermal ablation. Notably, the combination regimen incorporating the R837 adjuvant (Fe-PDA-PE-R837 + Laser) achieved the most potent therapeutic effect, with near-complete tumor regression, the smallest final tumor volume (Figure 5C), and the lowest tumor weight among all groups (*p* < 0.001 vs. Control group).

**FIGURE 5:**
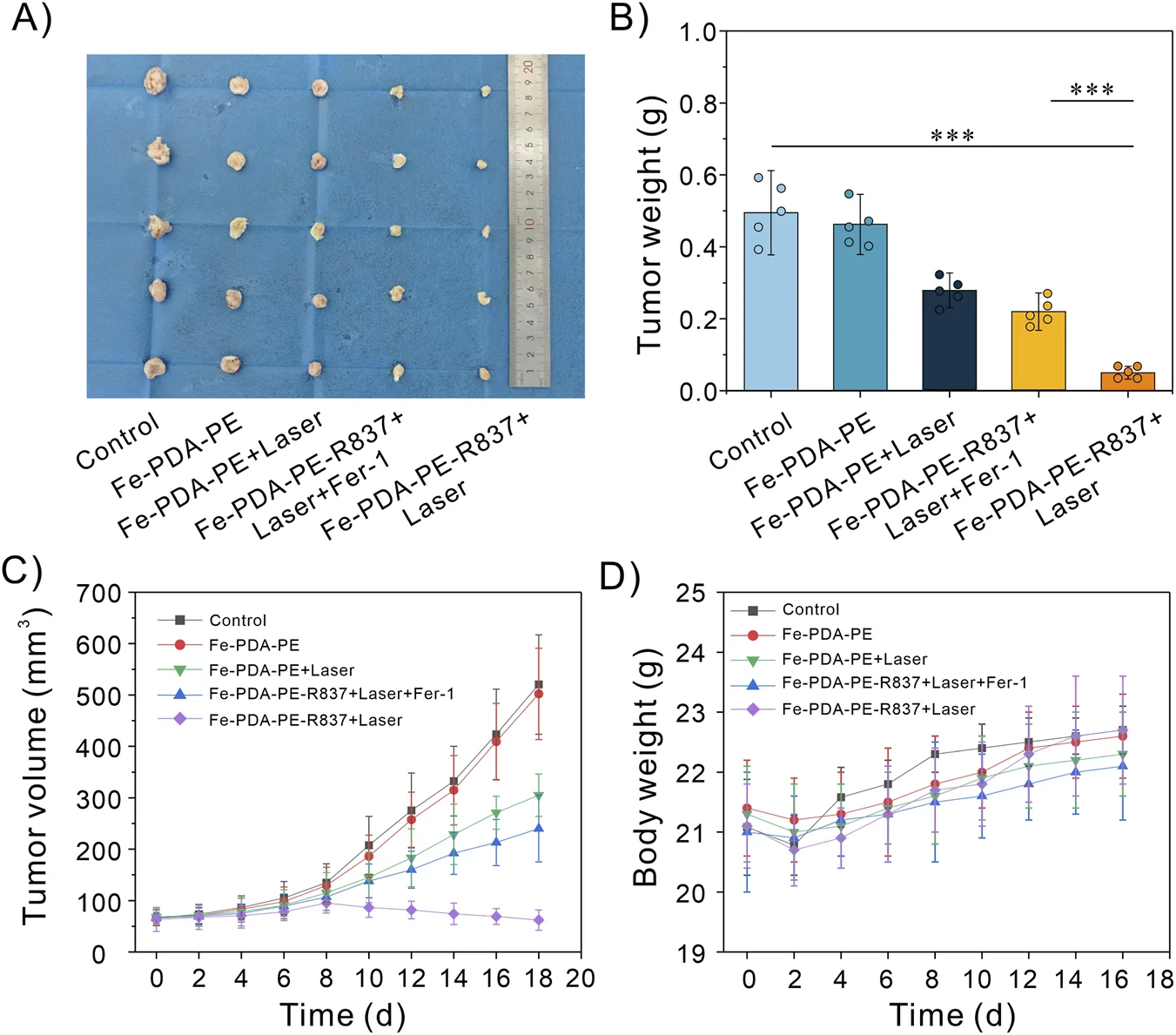
*In vivo* antitumor performance of Fe-PDA-PE-based nanotherapies. **(A)** Representative digital images of dissected tumors from each treatment cohort. **(B)** Statistical quantification of final tumor weights (***: *p* < 0.001). **(C)** Time-dependent tumor volume growth curves during the 18-day treatment cycle. **(D)** Body weight fluctuations of tumor-bearing mice across the whole therapeutic period.

To verify the functional contribution of ferroptosis to the observed antitumor effects *in vivo*, the ferroptosis inhibitor Fer-1 was co-administered with the combination treatment. As shown in Figure 5B, Fer-1 supplementation significantly attenuated the antitumor efficacy of the full combination regimen (p < 0.001, combination + Fer-1 vs. combination alone). This significant reversal of therapeutic efficacy confirms that ferroptosis induction is a core mechanism underlying the synergistic tumor suppression mediated by this nanoplatform.

Systemic biosafety was monitored throughout the 18-day treatment period via body weight tracking (Figure 5D). After a mild, transient weight fluctuation within the first 2 days of treatment, all experimental groups maintained steady body weight gain, with no significant difference from the control group. These results indicate that the Fe-PDA-PE-based nanomedicines and the applied laser irradiation have favorable *in vivo* biocompatibility with minimal systemic toxicity.

To elucidate the *in vivo* antitumor mechanism of Fe-PDA-PE-R837 plus laser irradiation, we characterized intratumoral iron deposition, ferroptosis-related gene expression, and both systemic and intratumoral immune cytokine levels across all treatment groups (Figure 6). First, ICP-MS analysis (Figure 6P) revealed the highest levels of both total iron and soluble labile iron (SLI) in tumor tissues from the Fe-PDA-PE-R837+ Laser group. The enriched intratumoral iron provides sufficient substrates for laser-triggered Fenton reactions, which generate toxic lipid peroxides and subsequently initiate ferroptosis cell death. Fer-1 co-treatment did not alter intratumoral iron accumulation but attenuated iron-mediated cytotoxicity, consistent with its role in targeting downstream lipid peroxidation rather than upstream iron metabolism.

**FIGURE 6:**
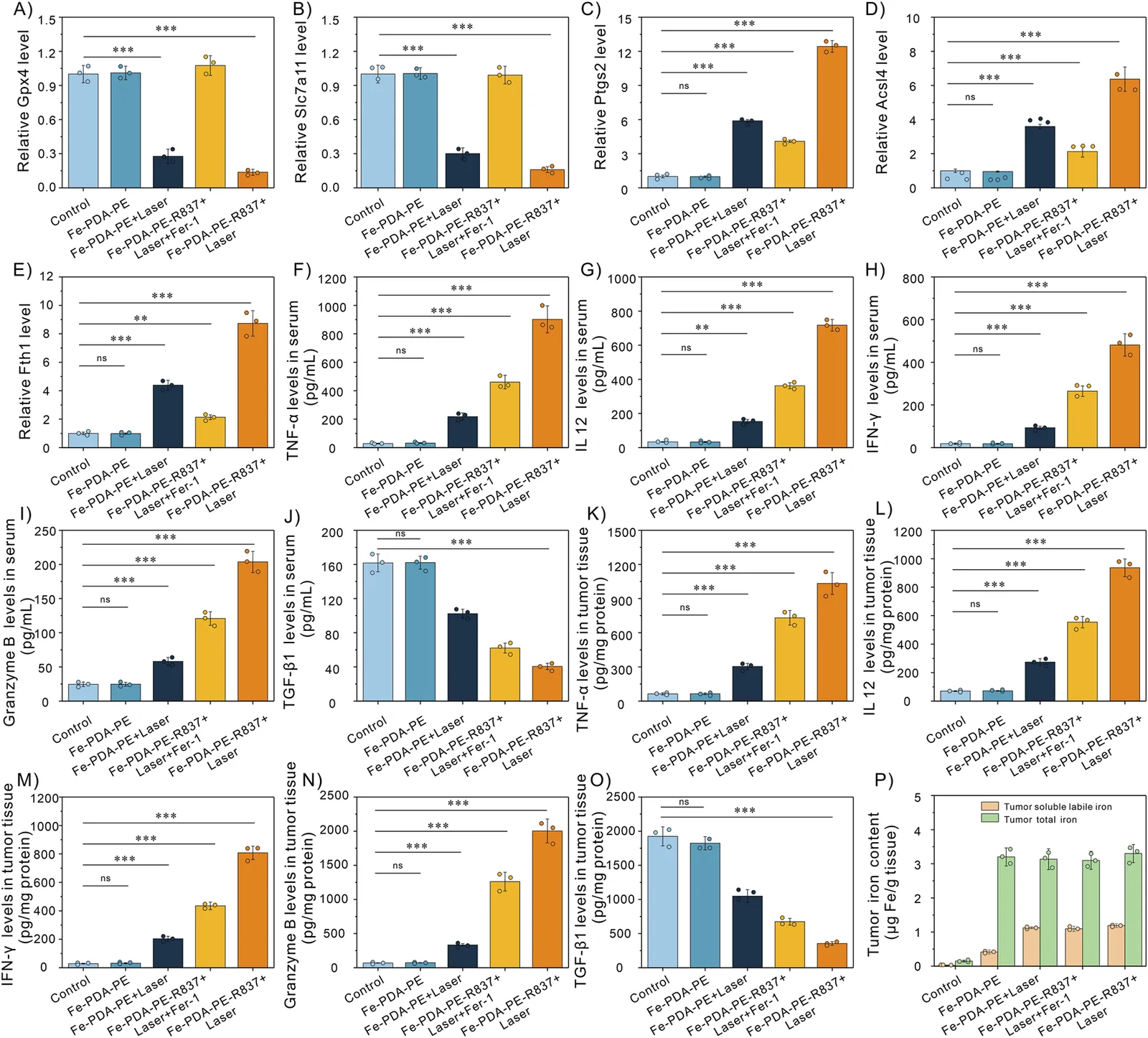
Detection of ferroptosis-related genes, serum and intratumoral immune cytokines, and tumor iron accumulation in 4T1 tumor-bearing mice after different treatments. **(A–E)** Relative mRNA expression levels of ferroptosis-associated genes *Gpx4*, *Slc7a11*, *Ptgs2*, *Acsl4* and *Fth1* in tumor tissues determined by RT-qPCR. **(F–J)** Concentrations of immune cytokines TNF-α, IL-12, IFN-γ, Granzyme B and TGF-β1 in mouse serum measured by ELISA. **(K–O)** Levels of TNF-α, IL-12, IFN-γ, Granzyme B and TGF-β1 extracted from tumor tissues quantified via ELISA. **(P)** Quantitative analysis of soluble labile iron and total iron content in tumor tissues detected by ICP-MS. Groups from left to right: Control, Fe-PDA-PE, Fe-PDA-PE + Laser, Fe-PDA-PE-R837+Laser + Fer-1, Fe-PDA-PE-R837+Laser. ns, not significant; ***p < 0.001.

Consistently, RT-qPCR analysis of tumor tissues (Figures 6A–E) demonstrated marked ferroptosis activation in the Fe-PDA-PE-R837+Laser group. Compared with the control, Fe-PDA-PE, and Fe-PDA-PE + Laser groups, the combination treatment significantly downregulated the anti-ferroptotic genes *Gpx4* and *Slc7a11*, while sharply upregulating the pro-ferroptotic genes *Ptgs2*, *Acsl4*, and the iron storage gene *Fth1*. These transcriptional alterations were largely reversed by Fer-1 administration, corroborating the synergistic ferroptosis-inducing effect of the Fe-PDA nanoplatform, R837 adjuvant, and laser irradiation (Yang et al., 2023).

We next quantified immune cytokines in serum and tumor homogenates to evaluate systemic and local antitumor immune responses (Figures 6F–O). Levels of Th1-type antitumor cytokines (TNF-α, IL-12, IFN-γ) and the cytotoxic effector molecule granzyme B increased in a treatment-dependent manner, reaching peak values in both serum and tumor tissues of the Fe-PDA-PE-R837 + Laser group. These cytokines support antitumor immune surveillance and direct elimination of malignant cells. In contrast, the immunosuppressive mediator TGF-β1 exhibited the lowest abundance in this combination group. Notably, blocking ferroptosis with Fer-1 markedly reduced pro-inflammatory cytokine secretion and partially restored TGF-β1 levels, indicating that ferroptotic cell death induced by our nanoregimen represents a potent form of ICD. Damage-associated molecular patterns (DAMPs) released during ferroptosis act synergistically with the R837 adjuvant to may contribute to remodeling the tumor immune microenvironment: they recruit effector immune cells, boost antitumor cytokine secretion, and alleviate TGF-β1-mediated immunosuppression.

Notably, the use of Pickering emulsion for R837 delivery is further supported by the work of Cao et al., who demonstrated that emulsion-based formulations of TLR agonists exhibited improved pharmacokinetic profiles and enhanced antitumor immunity compared to free drug (Cao et al., 2024). Our results are consistent with this finding, as the Fe-PDA-PE-R837 formulation showed minimal leakage under physiological conditions and rapid release in the acidic, reductive TME (Figures 2F,G), ensuring that R837 acts predominantly within the tumor microenvironment to stimulate local and systemic immune responses. This emulsion-based delivery strategy thus overcomes the rapid systemic clearance and poor tumor accumulation that typically limit the efficacy of free TLR agonists.

Compared with the control group, Fe-PDA-PE alone and Fe-PDA-PE + Laser treatments only induced mild ferroptosis and limited immune activation, indicating that iron-based photothermal monotherapy or dual therapy alone cannot trigger robust ferroptosis or potent antitumor immunity. Only the triple combination of iron-mediated photothermal ferroptosis and the R837 immunoadjuvant exerts dual synergistic effects: intratumoral iron overload drives extensive ferroptotic ICD to prime antitumor immunity, while R837 further amplifies Th1 immune responses for sustained tumor suppression. The Fer-1 rescue assays establish a direct causal link between ferroptosis and enhanced systemic and intratumoral immune responses, demonstrating that iron-dependent ferroptosis appears to serve as an important link connecting photothermal nanotherapy and immunotherapy.

### 3.6 *In vivo* systemic biosafety evaluation

To further valuate the systemic biosafety and translational potential of the Fe-PDA-PE-based nanotherapeutics, we performed histological and hematological assessments at the end of the 18-day treatment period. Major organs (heart, liver, spleen, lung, and kidney) were harvested from all experimental cohorts for hematoxylin and eosin (H&E) staining (Figure 7). Histological examination showed that the histological architecture of all treatment groups, including the full combination Fe-PDA-PE-R837 + Laser group, was comparable to that of the untreated control group. No evident inflammatory infiltration, cellular necrosis, architectural disruption, or apparent organ injury was detected, indicating favorable histocompatibility of the nanoplatform and the applied photothermal regimen.

**FIGURE 7:**
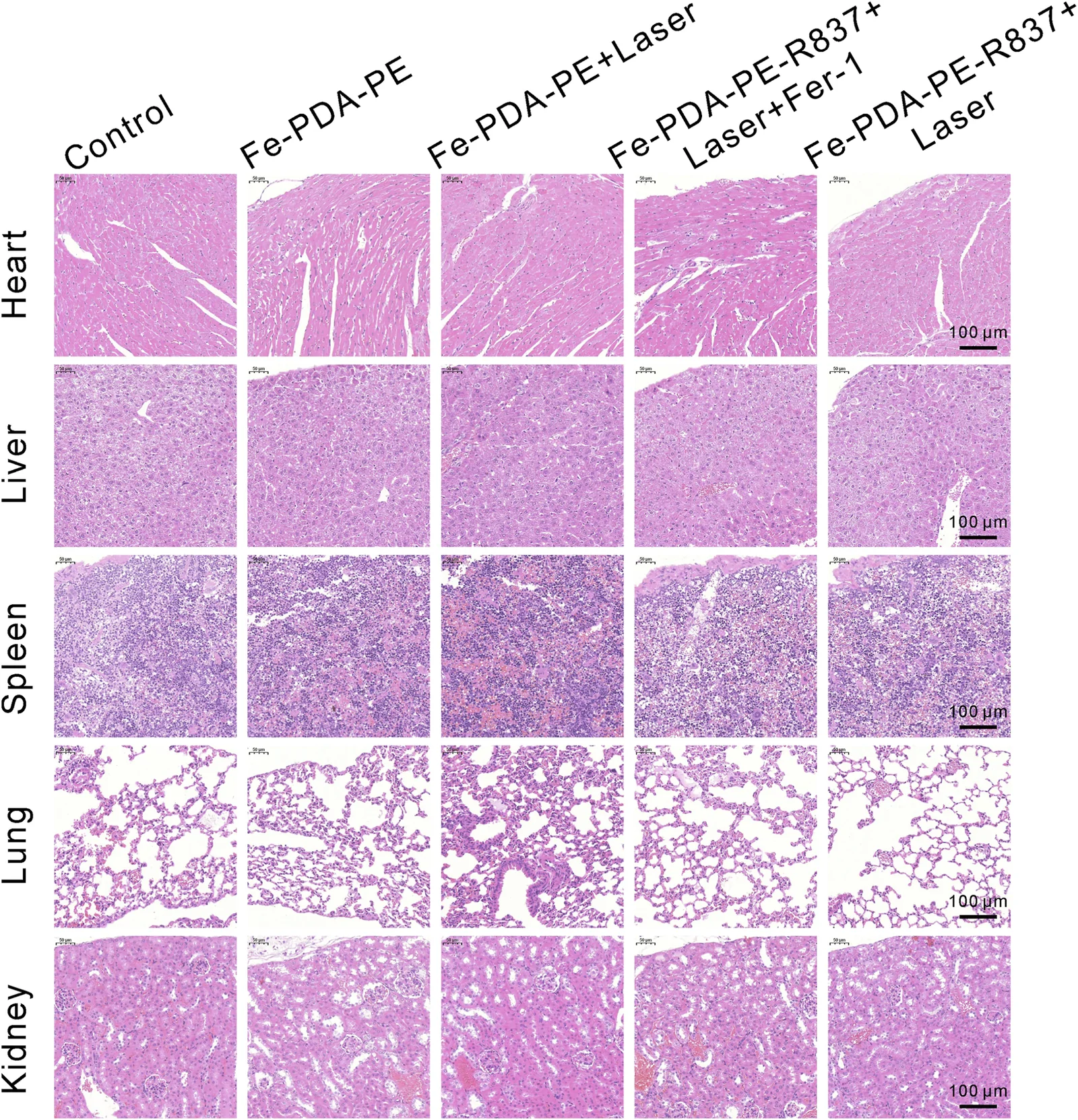
*In vivo* biosafety evaluation. Hematoxylin and eosin (H&E) stained images of major organs (heart, liver, spleen, lung, and kidney) collected from breast cancer-bearing mice after various treatments: Control, Fe-PDA-PE, Fe-PDA-PE + Laser, Fe-PDA-PE-R837 + Laser + Fer-1, and Fe-PDA-PE-R837 + Laser. Scale bars: 100 μm.

To further probe potential hematological and organ toxicity at the systemic level, blood samples were collected for complete blood count (CBC) and serum biochemistry analyses (Figure 8). The hematology panel (Figures 8A–C) showed that red blood cell (RBC), white blood cell (WBC), and platelet (PLT) count in all treated mice remained within normal reference ranges, with no statistically significant differences relative to the control group, indicating no evident hematological toxicity or myelosuppression. In addition, key biochemical markers of hepatic function (ALT and AST, Figures 8D,E), renal function (BUN and UA, Figures 8F,G), and cardiac function (CK, Figure 8H) remained within normal ranges with negligible fluctuations across all groups. Collectively, these histological, hematological, and biochemical findings demonstrate that the combination nanotherapy does not induce significant off-target toxicity, supporting a favorable safety profile and warranting further *in vivo* evaluation for translational applications.

**FIGURE 8:**
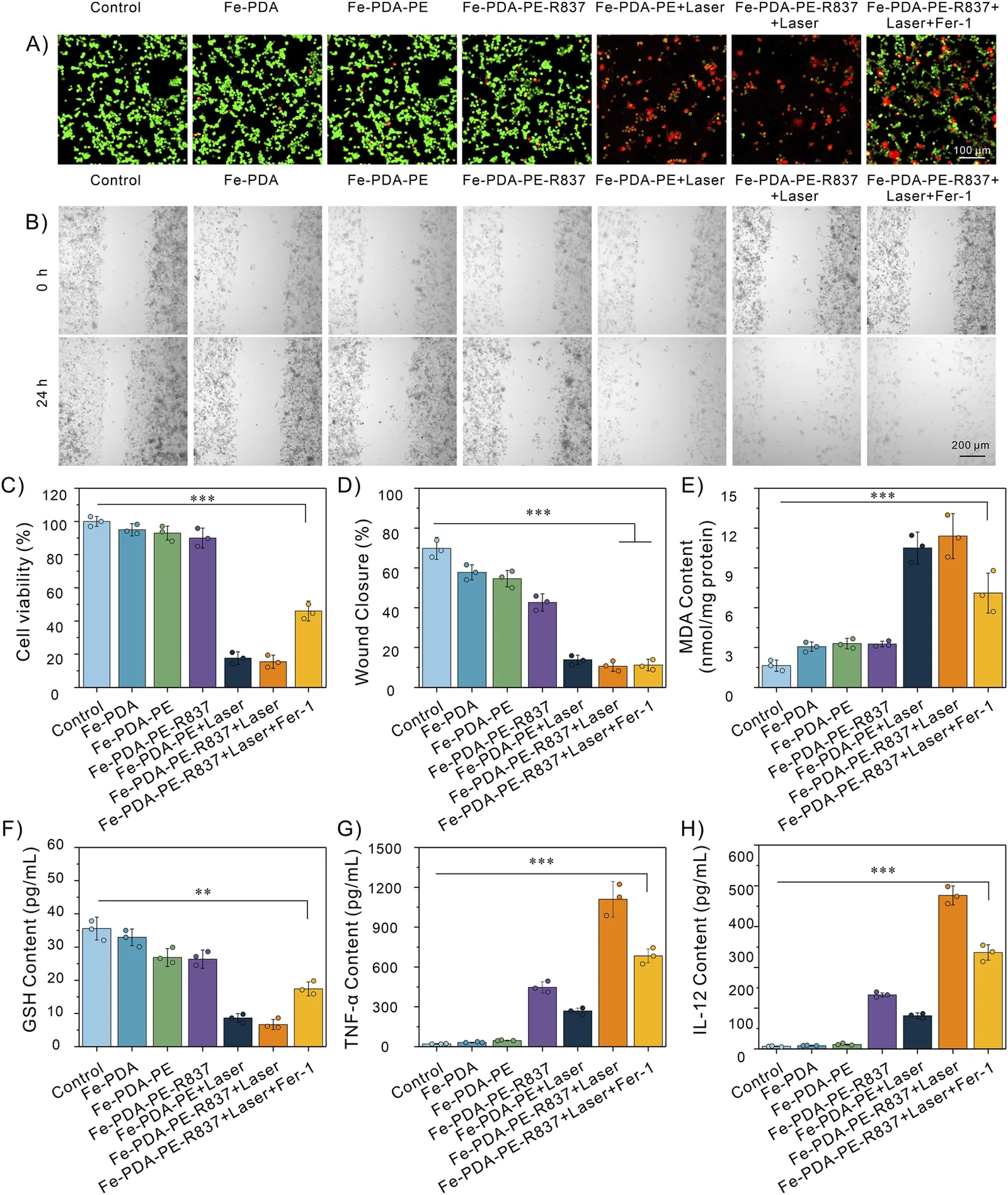
Blood hematology and biochemistry analysis of mice in different treatment groups. **(A)** Red blood cells (RBC), **(B)** White blood cells (WBC), **(C)** Platelets (PLT), **(D)** Alanine aminotransferase (ALT), **(E)** Aspartate aminotransferase (AST), **(F)** Blood urea nitrogen (BUN), **(G)** Uric acid (UA), and **(H)** Creatine kinase (CK). Groups from left to right: Control, Fe-PDA-PE, Fe-PDA-PE + Laser, Fe-PDA-PE-R837+Laser + Fer-1, Fe-PDA-PE-R837+Laser. Error bars represent standard deviations.

Despite the encouraging antitumor performance and biocompatibility profile of Fe-PDA-PE-R837, several limitations should be acknowledged. First, although ferroptosis involvement was supported by GSH depletion, MDA accumulation, and transcriptional changes in *Gpx4, Slc7a11, Ptgs2, Acsl4*, and *Fth1*, the current evidence remains largely correlative. More direct confirmation—such as lipid ROS detection by C11-BODIPY staining and protein-level validation of GPX4 and ACSL4 by Western blot—would strengthen the mechanistic conclusion. Second, the observed shift toward a Th1-skewed cytokine milieu suggests immune remodeling, yet definitive proof of ICD and systemic immune priming was not obtained. Key hallmarks of ICD, including surface calreticulin exposure, HMGB1 release, and ATP secretion, were not examined, nor was the infiltration and activation status of dendritic cells and CD4^+^/CD8^+^ T cells within the tumor. These experiments were beyond the scope of the present work but will be pursued in follow-up studies. Third, all *in vivo* efficacy evaluations were restricted to the 4T1 syngeneic breast cancer model. The translational relevance of this nanosystem would benefit from validation in patient-derived xenograft and large-animal models. Fourth, the biosafety evaluation was confined to an 18-day treatment window; long-term biodistribution, clearance kinetics, cumulative organ toxicity, and the durability of the elicited immune memory have yet to be determined. Addressing these gaps will be essential for advancing Fe-PDA-stabilized Pickering emulsions toward clinical application.

## 4 Conclusion

In summary, we developed an iron-doped polydopamine-stabilized Pickering emulsion (Fe-PDA-PE-R837) for localized triple-modal photothermal–ferroptosis–immunotherapy against triple-negative breast cancer, which overcomes the common limitations of premature drug leakage and low encapsulation efficiency of hydrophobic immunoadjuvants. Fe-PDA nanoparticles serve a dual role as both interfacial stabilizers and therapeutic functional components, integrating robust near-infrared photothermal conversion and tumor microenvironment-responsive iron release. The soybean oil inner core enables high loading of the hydrophobic adjuvant R837, while the compartmentalized emulsion structure allows spatiotemporally coordinated payload release. In the intratumorally treated 4T1 tumor model, 808 nm laser irradiation induces direct photothermal tumor ablation and accelerates labile iron release, thereby amplifying ferroptotic cell death. Ferrostatin-1 rescue assays verify that ferroptosis appears to play a pivotal role in bridging local photothermal therapy with the observed immune responses. Overall, this therapeutic regimen effectively suppresses primary tumor growth with a favorable safety profile, and further validation in large animal models is warranted to advance its clinical translation.

## Data Availability

The original contributions presented in the study are included in the article/Supplementary Material, further inquiries can be directed to the corresponding author.
