# Supplementary material for: Fe-doped polydopamine pickering emulsion as a nanoplatform for synergistic photothermal-ferroptosis immunotherapy in breast cancer
Source: Front Pharmacol. 2026 Oct 7;17:1956784. doi: 10.3389/fphar.2026.1956784 (PMC13685026; doi:10.3389/fphar.2026.1956784)
Supplement: Supplementary file 1 [file Supplementaryfile1.docx]

Supplementary Material

**Supplementary Figure 1.** UV-Vis standard calibration curve for R837 quantification.

**Supplementary Figure 2.** Linear calibration curve for iron element quantification by ICP-MS. X-axis: Fe concentration (μg/L); Y-axis: mass spectral signal intensity.

**Supplementary Figure 3.** Log-log standard calibration curve for sandwich ELISA quantification of mouse TNF-α.

**Supplementary Figure 4.** Log-log standard calibration curve for sandwich ELISA quantification of mouse IL-12.

**Supplementary Figure 5.** Log-log standard calibration curve for sandwich ELISA quantification of mouse IFN-γ.

**Supplementary Figure 6.** Log-log standard calibration curve for sandwich ELISA quantification of mouse Granzyme B.

**Supplementary Figure 7.** Log-log standard calibration curve for sandwich ELISA quantification of mouse TGF-β1.

**Supplementary Figure 8.** Zeta potentials of PDA and Fe‑PDA (PDA: -30.0±1.4 mV; Fe‑PDA: -20.9±1.8 mV).

**Supplementary Figure 9**. Water contact angle measurements of PDA (A, 55.67°) and Fe-PDA (B, 74.66°).

**Supplementary Figure 10**. Time-dependent changes in particle size and PDI of Fe-PDA nanoparticles incubated in saline solution over 7 days.

**Supplementary Figure 11.** Time-dependent changes in particle size and PDI of Fe-PDA nanoparticles incubated in serum medium over 7 days.

**Supplementary Figure 12.** Storage stability of Fe-PDA-PE-R837. The particle size was monitored for 5 days.

**Supplementary Figure 13.** Optimization of Fe-PDA-PE-R837. Encapsulation efficiency (orange) and volumetric loading amount of R837 (green) in emulsions prepared with different initial drug concentrations in the oil phase. All emulsions were fabricated at an oil-to-water volume ratio of 2:8.

**Supplementary Figure 14.** Volumetric Fe loading amount of Fe-PDA-stabilized Pickering emulsions prepared with different initial R837 concentrations in the oil phase.

**Supplementary Figure 15.** Hemolysis experiment photos of different concentrations of Fe-PDA-PE-R837.

**Supplementary Figure 16.** The UV–vis spectrum of the supernatant in the hemolysis experiment

**Supplementary Figure 17.** Hemolytic activity of Fe-PDA-PE-R837 at different concentrations.

**Supplementary Figure 18.** Intracellular total iron and soluble labile iron (SLI) levels in 4T1 tumor cells under different treatments. Total cellular iron was quantified by ICP-MS after complete acid digestion. SLI was separated from cell lysates using a 3 kDa ultrafiltration tube and determined by ICP-MS. All iron data were normalized to cell number and expressed as ng Fe per 10⁶ cells.

**Supplementary** **Table S1**. Hydrodynamic diameter and PDI of PDA and Fe-PDA nanoparticles measured by DLS from three independent experiments.

| Sample | Measurement | Hydrodynamic Diameter (nm) | PDI |
| --- | --- | --- | --- |
| PDA | Run 1 | 142 | 0.018 |
| PDA | Run 2 | 148 | 0.023 |
| PDA | Run 3 | 140 | 0.022 |
| PDA | Mean ± SD | 143.3±4.2 | 0.021±0.0026 |
| Fe-PDA | Run 1 | 164 | 0.031 |
| Fe-PDA | Run 2 | 171 | 0.026 |
| Fe-PDA | Run 3 | 156 | 0.029 |
| Fe-PDA | Mean ± SD | 163.7±7.5 | 0.029±0.0025 |
